# Insuring Value, Not Loss: Nursing Care, Accreditation, and Care-Pathway Performance in the Pricing of Private Health Insurance in Europe

**DOI:** 10.7759/cureus.115126

**Published:** 2026-08-24

**Authors:** Angela Greco, Giuseppe Fumai, Verdiana La Grotta, Gianluca Laricchia, Giuseppe Colonna

**Affiliations:** 1 Law, University of Bari Aldo Moro, Bari, ITA; 2 Administration, Private Insurance, Bari, ITA; 3 Nurse Quality Improvement, International Society for Quality in Health Care, Dublin, IRL; 4 Information, Communication, University Liaison and Training, Local Health Authority BT, Barletta, ITA; 5 Nursing, University of Foggia, Foggia, ITA; 6 Gynecologic Oncology, Cancer Institute "Giovanni Paolo II", Bari, ITA

**Keywords:** accreditation, clinical care pathways, economic value of nursing, health insurance premiums, health technology assessment, nurse staffing, private health insurance, quality-adjusted life year, quality indicators, value-based healthcare

## Abstract

Europe has entered a new era of health technology assessment. Clinical value is now appraised jointly across member states. Economic evaluation remains national. Cost-effectiveness, weighed in cost per quality-adjusted life year, is still decided country by country. Value is therefore appraised for what is used. It is not appraised for how well the care around it is delivered. Private health insurers face a parallel gap. The premiums they set rest largely on the characteristics of the insured and on claims history. They seldom reflect the quality of the care that members will actually receive. That quality is not intangible, as the evidence discussed here shows. Nurse staffing and nurse education are associated with hospital mortality. Structured clinical pathways are associated with fewer in-hospital complications and shorter stays. Accreditation is associated with a better safety culture and better organisational performance. The economic value of nursing has been quantified, where nurse-led care has been translated into savings and quality-adjusted life years. We therefore propose a heuristic model. Insurers would appraise these four elements periodically, in each contracted setting, and would let the result inform the premium. The aim is to reward the value that good nursing care creates. Today, the premium absorbs only the loss that poor care leaves behind. The model is a starting point. It would require actuarial modelling, piloting, and validation before use. It would also require safeguards against gaming and against penalising under-resourced providers.

## Editorial

Europe has entered a new era of health technology assessment (HTA); Regulation (EU) 2021/2282 is now applicable. Under it, a joint clinical assessment harmonises how the relative clinical effectiveness and safety of new health technologies are judged across member states. That single clinical appraisal then feeds national decisions [1]. Economic evaluation is different. It remains national. The cost-effectiveness analysis, weighed in cost per quality-adjusted life year (QALY), is still performed country by country [1]. Europe has therefore learned to appraise the value of what is used. It has not yet learned to appraise the value of how well the care around it is delivered.

Private health insurers face the same gap, and have more to lose from it. Our argument starts here from an observation of practice rather than from published evidence. The premium set for a health policy rests largely on the characteristics of the insured and on the claims those members go on to generate. It rarely reflects the quality of the care they will actually receive. Yet the insurer pays for that care. Every avoidable complication becomes a claim. So does every readmission. So does every unnecessary day in hospital. Premiums therefore look backwards, at losses already incurred. They do not look forward, at the care that decides whether loss occurs at all.

The care that decides it is, to a large degree, nursing care. A retrospective observational study examined 422,730 patients aged 50 years or older who had undergone common surgery. They were treated in 300 hospitals across 9 European countries. Each additional patient in a nurse's workload was associated with a 7% increase in the odds of dying within 30 days of admission (odds ratio 1.068, 95% confidence interval 1.031 to 1.106). Every 10% increase in the proportion of nurses holding a bachelor's degree was associated with a 7% decrease in those odds (odds ratio 0.929, 95% confidence interval 0.886 to 0.973) [2]. The authors then compared two staffing configurations. Patients in the better-staffed and better-educated one would have almost 30% lower mortality [2]. The design is observational, so these are associations and not proven causes. They remain, even so, the evidence on which any appraisal of nursing would have to begin.

This has an economic corollary. Nursing is one of the largest components of hospital operating expenses. Cutting it to save money may adversely affect patient outcomes [2]. The cheapest staffing is therefore not always the least costly. It can be the most expensive, once harm, prolonged stays, and readmission are counted. An insurer that ignores nursing when it appraises a provider is not being neutral. It is overlooking one of the strongest predictors of what it will have to pay.

The same holds for the pathways along which care is organised. A Cochrane review updated in 2025 included 58 studies, 24,841 patients, and 2,027 healthcare professionals [3]. Stand-alone clinical pathways likely reduce in-hospital complications from 17% to 10% (odds ratio 0.57, 95% confidence interval 0.41 to 0.80). The certainty of that evidence is moderate. They also likely shorten hospital stay by about 1.1 days. Costs and charges were generally lower where pathways were used, although the certainty there is very low. Pathways delivered as part of a multifaceted intervention gave mixed results, and the evidence about them is not conclusive. The settings tested were broad. They included general acute wards, emergency departments, intensive care, and extended-stay facilities. The conditions included asthma, stroke, mechanical ventilation, and myocardial infarction. The indicators used are the kind an insurer already records, namely, in-hospital complications, length of stay, readmission, and adherence to recommended practice [3]. Complications and days in hospital are not abstractions. They are the substance of a claim.

Organisational quality can be appraised too, and periodically. A systematic literature review screened 17,830 studies and analysed 76 empirical ones. It found reasonable evidence that compliance with accreditation standards brings multiple plausible benefits to hospital performance [4]. The details matter for an insurer. Accreditation showed a consistent positive effect on safety culture, process-related performance measures, efficiency, and patient length of stay. It was unrelated to employee satisfaction, patient satisfaction and experience, and to the 30-day readmission rate. Its effect on mortality and on healthcare-associated infections was paradoxical, so no firm conclusion could be drawn there. A further finding deserves attention. The effect on healthcare workers, and on job stress in particular, was the exception to the positive pattern [4]. Accreditation therefore moves some of what an insurer pays for, and not all of it. That is an argument for combining it with other measures rather than leaning on it alone. It remains periodic, externally verified, and already comparable between providers.

What remains is the part of nursing that the financial reckoning leaves out. The economic value of nursing has been defined as the return on investment in nursing human capital, visible in patient, organisational, and workforce outcomes [5]. Nursing is nonetheless still classified as an operational cost rather than as a driver of clinical and economic value. Emotional labour, which sits at the centre of the discipline, is overlooked because it is less visible and harder to quantify. The consequence is not only cultural. Evidence linking nursing interventions to better outcomes often fails to be translated into monetary terms, and no standardised economic framework exists to perform that translation. Where the translation has been made, the figures are substantial. A nurse-led initiative that cut central line-associated bloodstream infections by 40% saved two million dollars a year. Community nurse-led education for patients with heart failure in Italy cost 1.3 million euros more and produced 247 additional QALYs [5]. The unit Europe uses to appraise its technologies has therefore already been used to appraise nursing care. It has not yet been used to price it.

Four appraisable things therefore exist. Nurse staffing and nurse education are quantified and linked to mortality [2]. Care-pathway performance is quantified and linked to complications, length of stay, and cost [3]. Accreditation is verified periodically and linked to safety and organisational performance [4]. The economic contribution of nursing has been quantified where it has been translated into monetary and QALY terms, and its inclusion in value-based models has already been proposed [5]. We are not aware that private insurers appraise any of them when they set a premium. Economic evaluation of technologies has a common unit in the QALY [1]. Nursing care has no comparable unit in the premium. We suggest that one could be built, and that the discipline already established for technologies offers a method [1].

We offer the following as a heuristic model rather than a finished tariff. It sets out five domains. For each domain, it names what an insurer could appraise, periodically and setting-by-setting, and how the result could inform the premium. The model is deliberately a starting point. It would be modelled, piloted, and revised rather than adopted as it stands. We call it a model for insuring value, not loss (Table 1).

**Table 1:** A heuristic model for insuring value, not loss: five domains by which private health insurers could appraise nursing care, care-pathway performance, and accreditation, and let them inform the premiums set for health policies. Authors' own elaboration, offered as a heuristic model and a starting point rather than a finished tariff. The elements to be appraised are drawn from the cited evidence. Each domain would require actuarial modelling, local piloting, and validation before use.

Domain	What an insurer could appraise, periodically and by setting	How it could inform the premium (staged and adaptable)
1. Nurse staffing and education	Patients per nurse. Proportion of nurses holding a bachelor's degree. Recorded in each contracted setting.	Two determinants of mortality are already quantified [2]. The published odds ratios could seed a first actuarial calibration.
2. Care-pathway performance	The use of structured clinical pathways and their indicators, namely, in-hospital complications, length of stay, readmission, and adherence to recommended practice [3].	Reward the pathways that lower complications and shorten stays, since these are where claims are generated. Extend the same indicators to outpatient and community settings.
3. Accreditation and quality requirements	Accreditation status. Periodic verification of quality requirements in each contracted setting.	Treat externally verified organisational quality as a comparable signal that could modulate the premium, and reverify it at each renewal. Do not lean on it alone, since accreditation appears unrelated to readmission and to patient experience [4].
4. Economic translation of nursing care	Whether nursing-sensitive outcomes in each contracted setting are translated into economic terms, in savings or in QALYs [5].	Weigh what nursing generates and not only what it costs. The translation has already been performed for nurse-led initiatives, and the inclusion of nursing metrics in value-based models has already been proposed [5]. The premium is where a payer could apply it.
5. Transparency and revision	The published basis and weighting of each premium. The outcomes that matter to the insured.	Make the weighting explicit. Revise it as evidence accumulates. Guard against gaming, and against penalising under-resourced providers.

One feature of the model deserves emphasis because it is what would make a start possible. Part of it is already quantified. The association between nurse staffing, graduate education, and 30-day mortality has been estimated as odds ratios [2]. The effect of clinical pathways on complications and length of stay has been estimated with moderate certainty [3]. Those estimates could seed a first actuarial calibration. It would then be tested and refined against an insurer's own claims data. Accreditation is already verified on a cycle [4]. The economic translation of nursing outcomes has already been performed on a case-by-case basis [5]. A model that begins from what is already measured seems easier to propose than one that waits until everything is.

The obstacles are real, and worth naming. Actuarial models are built on loss data. Translating pathway performance or the economic contribution of nursing into premium terms would demand new metrics and careful validation. The underlying evidence is graded moderate rather than high [3]. The effect of accreditation varies widely between settings and outcomes [4]. Data are fragmented across settings and providers. Perverse effects are possible. Any scheme would need safeguards against gaming. It would also need safeguards against penalising providers that serve the most difficult populations with the fewest resources. These are reasons for caution and for modelling. They are not reasons to keep appraising safety as though it did not exist.

Europe has decided to appraise the value of its technologies together; and the HTA era is proof of it [1]. The harder step would be to appraise the value of its care; it may also be the more valuable one. Private insurers are unusually well placed to take it because they already pay for the consequences of care they never examine. The proposal is simple in principle. An insurer could come to reward the value that good nursing care creates, and not merely absorb the loss that poor care leaves behind.
